# Biotech and Biomaterials Research to Reduce the Caries Epidemic

**DOI:** 10.1186/1472-6831-6-S1-S1

**Published:** 2006-06-15

**Authors:** Rebecca L Slayton, James D Bryers, Peter Milgrom

**Affiliations:** 1 Department of Pediatric Dentistry, University of Washington, Seattle, WA 98195, USA; 2 Department of Bioengineering and University of Washington Engineered Biomaterials (UWEB) Center, University of Washington, Seattle, WA 98195, USA; 3 Department of Dental Public Health Sciences, University of Washington, Seattle, WA 98195, USA

## Abstract

The goal of this workshop is to develop a consensus within the biomaterials/bioengineering community for a research agenda focused on creating technologies that will address the current dental caries pandemic. The workshop will bring together expertise from academia, industry, and the NIH institutes in the areas of oral biofilm microbiology and innovative biomaterials. The rationale for the workshop is that science and technology have not produced sufficient practical tools for public health practitioners and the private delivery system to address the pandemic in dental caries that exists for children and adults from families with low incomes and for numerous ethnic minority and racial groups. Moreover, it is unclear whether the barriers are remediable bioengineering and technical problems or fundamental science questions. Nevertheless, the obligation to address the gap between scientific research and practical application is especially relevant today. The U.S. and state governments bear the majority of the cost of trying to control this pandemic through Medicaid, the Public Health Service, Indian Health Service and other similar programs. These costs continue to escalate as continued applications of existing technology are unlikely to markedly reduce disparities. The mainstays of caries prevention, topical and systemic fluorides and pit and fissure sealants, are technologies developed in the 1950s and 1960s.

## Introduction

The caries process has been well understood since 1960 when Fitzgerald and Keyes demonstrated in animals that acidogenic bacteria are transmitted from mother to offspring and between animals sharing the same cage [1]. These bacteria metabolize carbohydrates and produce acid, which in turn leads to demineralization of dental enamel. Dental caries is thought to be an almost completely preventable disease. Prevention requires minimizing the frequency of ingesting simple carbohydrate foods and beverages, regular oral hygiene measures to remove plaque and to introduce topical fluoride in the form of toothpaste, and flossing to remove plaque and debris from between the teeth. It is recognized that even when the appropriate preventive measures are taken, there are some individuals who have increased susceptibility to dental caries.

At a group level, people living in poverty, ethnic and racial minorities, and recent immigrants to the U.S. are at increased risk for dental caries. There is also evidence that people with poor access to dental care are at increased risk. In children, dental caries is the most common chronic illness and is five times more common than asthma [2].

The discovery of the role of fluoride in dental caries prevention led to significant decreases in caries prevalence in the U.S. and many other countries as a result of community water fluoridation and the widespread use of fluoridated toothpaste [3]. At the turn of the current century, the U.S. Centers for Disease Control and Prevention determined that water fluoridation was one of the top 10 public health achievements of the century [4].

In the last decade, there has been an increased interest in research related to microbial biofilms as well as in bioengineering and biotechnology. It has become increasingly evident that dental researchers need to look beyond their traditional mind set and investigate the opportunities for collaborating with researchers outside of dentistry in order to bring new insights to the prevention and management of a disease that continues to affect a large percentage of the population in all countries of the world.

This need for a new, innovative approach to the management and prevention of dental caries resulted in the National Institute for Dental and Craniofacial Research (NIDCR) and others providing funding for an interdisciplinary conference on biotechnology, biomaterials, and dental caries (Dr. Peter Milgrom, P.I.). The purpose of this conference was to develop a research agenda that will leverage new discoveries and lead to innovative approaches to reduce caries in high risk populations. An esteemed group of internationally recognized scientists representing cariology, behavioral sciences, bioengineering, and microbiology were invited to present research on topics aimed at developing a better understanding of this multifactorial disease. Emphasis was placed on the development of interdisciplinary collaboration between dentistry and bioengineering.

There were 57 participants including 34 nationally and internationally known scientists, four dental practitioners, four representatives from the dental industry and 15 graduate and undergraduate students. The meeting was spread out over two and a half days with four plenary sessions and three breakout sessions.

Two keynote lectures were given during lunch on the second day and breakfast on the third day, respectively. The first lecture was given by Pierre Mourad, PhD, Principal Physicist, Applied Physics Laboratory, Research Associate Professor, Department of Neurological Surgery, University of Washington School of Medicine and was entitled "Translational Medical Research: Examples and Lessons." Dr. Mourad discussed important considerations when moving a research method or device from the laboratory into market. Since he has gone through this process with a number of his own inventions, he was able to provide valuable tips to the participants.

The second lecture was given by Christopher J. Elias, MD, MPH – President, Program for Appropriate Technology in Health (PATH). His talk was entitled "Public-Private Partnerships to Advance Technologies for Neglected Diseases." In his talk, Dr. Elias emphasized that partnerships with private companies must be in keeping with the mission of PATH and must "lead to positive impact on availability, accessibility, and affordability of important health products for public health programs in developing countries." He also pointed out the importance of recognizing the need for profitability for the private company and that "PATH must recognize the company's need for commercial benefit in order to ensure a sustainable commitment to the collaboration." He used the example of the meningitis vaccine project in which PATH, with a grant from the Bill and Melinda Gates Foundation, partnered with the World Health Organization and an emerging vaccine manufacturer in India to produce an affordable vaccine for use in Sub-Saharan Africa. In this initiative and in many others, PATH has demonstrated that public-private partnerships are an effective means to advance technologies to treat neglected diseases. This is particularly relevant for dental caries since it is prevalent in both developed and underdeveloped nations and frequently affects individuals with the least ability to access limited resources.

It was clear from the presentations at this conference that there have been major advances in both biotechnology and bioengineering in the last decade. The themes that presenters were asked to address were divided into three general categories: 1) ways to modify or enhance existing technologies (fluoride- or xylitol-related) to make them more effective; 2) development of new targets or novel strategies (related to adhesion, oral ecology, or probiotics); 3) development of new technologies or delivery systems (related to anti-adhesives or fluoride replacement methodologies).

Presenters discussed emerging research in the prevention of dental caries including: specific species bacterial adhesion prevention, biofilm ecology manipulation, targeting of microbial species using lytic enzymes, probiotics using natural anti-microbial peptides, circumvention of sucrose-driven acid production, and fluoride- and other antimicrobial-release coatings. These topics were the focus of discussion during the conference and informed the breakout sessions each day.

### Challenges to Participants

The conference opened with remarks from Dr. Peter Milgrom, followed by a series of talks that described the challenges faced by clinicians and researchers in their efforts to minimize the impact of dental caries. Dr. Burton Edelstein discussed the term "pandemic" and the appropriateness of using this term to describe dental caries. He made the point that use of the term "caries pandemic" suggests a disease that is highly prevalent globally and has severe consequences to society. He also initiated the discussion of caries being a disease that is amenable to prevention and that is experienced disproportionately within the population, with poor and disadvantaged people being more frequently affected. This theme was continued and refined by Dr. Clemencia Vargas who defined health disparities as the occurrence of a disproportionate share of the burden of health being received by one particular group of people. She went on to describe Early Childhood Caries (ECC) as a disease with disparities in both the prevalence and treatment. Dr. Vargas presented compelling data to show that when white children are compared to non-white children in the U.S., non-white children have a greater caries experience and a higher level of untreated caries.

Dr. Donald Patrick presented a theoretical framework for understanding and investigating disparities in oral health. This model, which discusses the various factors that contribute to oral health and disease and identifies interventions that target each of these factors, will bring new direction for research and policy that is focused on reducing oral health disparities.

Evidence shows that education alone is not sufficient to change behavior. Since a significant component of dental disease is related to behaviors such as diet, oral hygiene, and fluoride exposure, it makes sense that any strategy to improve oral health must improve health-related behaviors. Dr. Philip Weinstein's presentation on motivational interviewing as a technique to assess a person's readiness to change and to subsequently effect that change, was an important reminder to all participants that any solution to dental caries must consider adherence or lack of adherence by individuals. He also discussed this same theory relative to the practitioner's readiness to change, as frequently there are effective methods to prevent dental caries that dentists choose not to incorporate into their practices.

The final presentation in this section was by Dr. Joel Berg and was focused on the opportunities for developing and marketing new technologies for caries prevention. Dr. Berg emphasized the importance of knowing the market and identifying unmet needs on the part of both the buyer and seller. Once this is accomplished, then the market should be divided into segments, and the particular segment of interest can be targeted. The dental market in the U.S. is approximately $80 Billion, and 20 percent of this market is for consumer or professional products. In terms of categories of expenditures, 60 percent of the total expenditures are for either the results or effects of dental caries. As caries detection technologies become more sensitive, there will be greater opportunities to manage the caries process (to remineralize the enamel surface) rather than to intervene surgically. In general, the market for new products (such as early detection devices) is very difficult. The marketplace will ultimately not be receptive to these newer highly sensitive tools, even with increased specificity, unless the appropriate compensation systems are in place.

### Delivery Challenges for Caries Preventive Agents

Caries preventive agents, such as fluorides and chlorhexidine, have been shown to be very effective at remineralizing surface enamel or controlling cariogenic bacteria, respectively. However, both of these agents rely on patient compliance at home or regular visits to the dentist. Recent studies also show promise for other agents such as xylitol (a sugar alcohol) and amorphous calcium phosphate in toothpaste and chewing gums. All of these agents require action on the part of the patient in order to have the desired effect. Some are not particularly palatable. That is, the characteristics of both the agent and the delivery system have an impact on its effectiveness, and current approaches might be redesigned to advantage.

The first plenary session of the conference focused on delivery challenges associated with proven preventive agents. In his talk, Dr. John Featherstone discussed the shortcomings of current delivery methods, including the short duration of therapeutic levels of fluoride and chlorhexidine when administered as a varnish or rinse and the need for ways to deliver these agents over an extended period of time. Xylitol has been shown to be effective when consumed as a gum or lozenge, but neither of these delivery methods is appropriate for an infant. In his presentation, Dr. Featherstone argued for research focusing on strategies that combine agents that would be desirable because the effectiveness of fluorides in remineralization would be enhanced if the bacterial load were reduced first. The traditional delivery mechanisms for fluorides and other remineralizing agents have been in dentifrices and mouth rinses. Dr. Domenick Zero discussed the regulatory issues related to getting approval for adding new cariostatic agents to toothpaste or mouth rinses as they are tested and found to be effective. Dr. Zero identified a number of factors that interfere with the development of the most effective caries preventive agents for the U.S. including: 1) U.S. Food and Drug Administration guidelines that focus on safety and efficacy (not level of efficacy); 2) rising costs of clinical trials; 3) Laboratory testing requirements that use antiquated methods which no longer reflect our current understanding of the action of fluoride. In his paper, he recommends that oral health disparities can be addressed by making certain that at-risk individuals are obtaining the maximum benefit from fluoride dentrifice by making available the most effective products and educating individuals on how to obtain the greatest benefit.

Dr. Per Axelsson reported the results of a 20-year study that implemented a caries-preventive program for children in different age groups or developmental stages. Risks were associated with initial acquisition of *mutans streptococci *in infants, eruption of first permanent molars in 5–7-year-olds, and eruption of second permanent molars in 11–13-year-olds. Individual risk for caries was determined annually, and those children considered at high risk were given additional preventive interventions including fluoride varnish application and more frequent professional mechanical tooth cleaning. This program has been very successful in reducing the caries experience in children in Varmland, Sweden. Among 19-year-olds cross sectionally, the average caries prevalence was reduced from more than 24 DFS in 1979 to only 2 DFS in 1999. This study is an example of how our current technologies, applied intensively on a risk basis in schools and public dental health centers and timed to coincide with the eruption of new, susceptible tooth surfaces, can be very effective at reducing the burden of disease.

### Dental Caries Microbiology

Very early in the study of dental caries (in the late 19^th ^century), W.D. Miller recognized that enamel demineralization was mediated by a mixed bacterial infection in whole saliva [5]. What he didn't know was which bacterial species were responsible for metabolizing carbohydrates, converting sugar to acid and leading to demineralization of dental enamel. The identification of *Streptococcus mutans *as a possible caries-causing agent occurred much later [1] and has since been expanded to include other *Streptococci *species, referred to collectively as *mutans streptococci *[6]. Caries research since that time has focused primarily on *mutans streptococci *as a target for prevention of disease through the use of anti-microbial agents, vaccines, and probiotics.

The second plenary session focused on new technologies and approaches used to understand the microbiology of dental caries and ways to interfere with the caries process. Dr. David Stahl discussed the composition of the oral biofilm and the evidence that this biofilm is inhabited by more than 600 microbial species. Less than half of these bacteria are cultivatable by traditional means. Dr. Stahl's laboratory uses microchip technology to identify bacteria within the biofilm and to determine their relative frequency within plaque samples.

Dr. Howard Kuramitsu discussed the importance of interactions between *S. mutans *and other biofilm constituents in determining the cariogenicity of plaque samples. In particular, his laboratory investigated the ability of *S. gordonii *to weaken some of the properties of *S. mutans *that contribute to its virulence, including its ability to produce bacteriocin, its ability to form a biofilm, and its ability to transform its genetic makeup. Research regarding these strategies, he argued, may be valuable in findings methods to control or manage dental caries in some individuals. Additional strategies were suggested by Dr. Phil Marsh, who focused on the importance of understanding oral ecology and the factors that disrupt microbial homeostasis as an essential part of caries control mechanisms. He asserted, in his lecture, that it is necessary to identify critical control points on an individual basis in order to interfere with the disease process rather than just treating the consequence of disease.

Research on oral biofilms is essential to the understanding of dental caries. Biofilms are composed of multiple bacterial species that adhere to a solid surface (the tooth) and are held together by a matrix of extracellular polymeric substances (EPS). Characteristics of biofilms that make their control challenging include the increased resistance to antimicrobial agents of the bacteria within the biofilm and the rapidity of re-colonization after mechanical removal. Dr. John Cisar presented research from his lab regarding the bacteria that initiate colonization of tooth surfaces. His lab identified streptococcal receptor polysaccharides (RPS) that interact with viridans group *streptococci *and *Actinomyces naeslundii *in early biofilm formation. Understanding this important component of the biofilm and how it differs from one individual to another may provide new insights and through research lead to new approaches to the management and prevention of dental caries.

The recognition that antimicrobial peptides are present in saliva provides some interesting possibilities for the management of oral diseases such as dental caries and periodontal disease. Dr. Beverly Dale-Crunk measured levels of antimicrobial peptides in school-aged children with or without dental caries. In her study, she found that mean levels of alpha-defensins were significantly higher in some children without caries than in other children with caries. These results, she argues, suggest possibilities both for early detection of caries risk and for early preventive strategies through research.

### Emerging Technologies

Technologies with the potential to prevent or manage dental caries could take a number of forms, including ways to detect caries risk prior to disease occurrence, species-specific targeting of acidogenic bacteria, reduction of bacterial adherence to tooth surfaces, and prolonged release of therapeutic agents. Interdisciplinary research that combines the expertise of dentistry, molecular biology, and bioengineering holds great promise for new, innovative approaches to the prevention of dental caries.

Dr. Vincent Fischetti presented a study on the use of bacteriophage lytic enzymes to control pathogenic bacteria. These enzymes are species specific and are capable of destroying large numbers of bacteria in blood or on mucosal surfaces in seconds. They target the bacterial cell wall of the specific bacteria they were developed from and do not attack human cells. Lytic enzymes have many potential uses in the food industry, in hospitals and nursing homes, and in the treatment of diseases such as dental caries that are mediated by bacteria. He suggested that an enzyme specific to *S. mutans *is being developed.

One of the key ingredients in the caries process is diet. A diet rich in sugars and simple carbohydrates provides the oral bacterial with a substrate from which they produce acid. Recent evidence suggests that a person's preference for sweet-tasting foods is more complex than simply what foods or sweets the child is exposed to early in life. Dr. Danielle Reed demonstrated in her work that sweet perceptions and preferences have an important genetic component, suggesting that some people may actually have a "sweet tooth." Having the genetic makeup that leads to a preference for sweet-tasting foods does not necessarily predict the choices an individual will make. It does, however, suggest another potential avenue for the investigation of risk factors leading to dental caries.

Technologies to control biofilm formation have been developed in many disciplines other than in dentistry. Management or elimination of microbial biofilms is important in fields as diverse as medicine and the boating industry. In the boating industry the management of biofilms has led to the development of anti-fouling polymers such as poly(ethylene glycol) (PEG), or a poly(N-substituted glycine) (polypeptoid). Dr. Phil Messersmith discussed his research with these polymers and how these technologies may have interesting possibilities for the management of oral biofilms and dental disease.

Dr. Buddy Ratner expanded on the topic of anti-adhesive polymers and discussed a number of other strategies that have potential with future research for disrupting the oral biofilm or delivering therapeutic agents in a controlled-release manner. The controlled delivery systems he discussed include passive delivery systems and responsive drug delivery systems. Examples of the passive systems include diffusion-controlled systems wherein the drug is either in a reservoir device surrounded by an inert barrier or membrane or the drug is dissolved in an inert polymer. Another type of passive system is chemically controlled. A bioerodible system is used to hold and subsequently release the drug. Responsive drug delivery systems are either open- or closed-loop systems. Open-loop systems are regulated externally using magnetism or ultrasound. In closed-loop systems, the release is in response to local conditions such as temperature, pH, or other factors. Advances in understanding of the caries process and of the many new engineered biomaterials has provided many future opportunities for collaborative efforts aimed at eliminating dental caries in the most susceptible individuals.

### Future Goals

One of the stated goals of this conference was to develop a research agenda that, if adopted by investigators and funders, will leverage recent discoveries and lead to innovative new approaches to reduce caries in high-risk populations. To allow discussion leading to a research agenda, there were three breakout sessions with three sub-topics in each session. Participants were charged with identifying 1) ways to enhance existing technologies; 2) new targets or novel strategies and 3) new technologies or delivery systems to decrease the incidence of dental caries. Strategies identified in the breakout sessions were then organized into specific topics that could inform a future request for applications from government, foundations, or industry in the area of biotechnology and bioengineering approaches to reduce disparities in dental caries.

Studies that would be responsive to the goals of this RFA would include but not be limited to the following:

### Enhancing Existing Technologies

• Methods to improve the substantivity of topical fluorides; techniques to enhance the diffusion of fluoride into plaque; innovative, controlled release delivery devices; combinations of fluoride and antimicrobial therapies; and high-concentration fluoride varnishes.

• Education programs to inform the public and the health care community about disease management and prevention using psychological approaches, including motivational interviewing and other approaches used effectively outside the field of oral health.

• Clarification and enhancement of mechanisms of action v/v inhibition of acid production and glucan production.

• Development of toothpaste with fluoride encapsulated in controlled-release "microcapsules" substantive to plaque, or pellicle/tooth surface.

• Formulation of toothpastes with complimentary agents such as lytic enzymes or anti-microbial peptides.

• Incorporation of novel technology such as quorum sensors, adherence inhibitors, bacterial toxins, and polymer-releasing salicylic acid derivatives.

### Development of New Targets or Novel Strategies

• Methods to effectively balance the oral ecology through the delivery and retention of agents such as antimicrobial peptides, arginine, urea, and fluoride.

• Methods to identify the full range of oral bacteria and understand the metabolic activity of acidogenic species.

• Methods to recognize and inhibit virulence factors.

• Methods to inhibit metabolic pathways and to deliver and retain buffers in plaque.

• Development of delivery systems that can be done at home – rinse, brush or wipes.

• Enhanced production of antimicrobial peptides.

• Development of probiotic approaches such as introducing *S. gordonii *prior to establishment by *Mutans streptococci*.

• Development of novel methods to target *Mutans streptococci *such as lytic enzymes, competence-stimulating peptide (CSP), and mutacin.

• Identification and development of lytic enzymes to target other acid producing oral bacteria.

• Identification of biomarkers for inhibitors of quorum sensing and for inhibitors of bacterial metabolism.

• Development of new delivery systems to provide for distribution throughout the mouth and release over extended periods of time.

• Development and testing of antiadhesive coatings for controlling oral biofilms, including research to define the chemical and structural characteristics desired in antifouling polymers.

• Development and testing of the chemical and structural characteristics of glycoproteins and polysaccharides present in oral biofilms.

### Development of New Technologies or Delivery Systems

• Modification of xylitol delivery systems to reduce the number of doses while maintaining its effect.

• Research to understand how xylitol is affecting the bacterial ecology in plaque and saliva.

• Development and testing of the efficacy of sustained release of xylitol.

• Testing the ability of small molecular-weight antimicrobial compounds (peptides, etc.) tethered to antifouling polymers to be active in controlling bacterial colonization and biofilm formation.

• Evaluation of the behavior of microorganisms in contact with antifouling polymer coatings.

• Evaluation of the ability of antifouling polymer coatings to be combined with release of active compounds (fluoride, chlorhexidine, etc.) to more effectively treat/prevent caries. Possibilities include entrapping nanoparticles within coatings for long-term release of antibacterials.

• Testing the ability to design antifouling polymers that encourage colonization of tooth/device surfaces by "good" microorganisms over "bad" (acid secreting) microorganisms.

• Development of methods to improve the residual capacity of fluoride delivery including sustained-release materials, "responsive" release materials (e.g. pH-triggered), combination therapy (antibiotic/fluoride) release, and biomaterials that buffer pH.

## Conclusion

Successful outcomes of this conference include the promotion of a dialogue between end-users, oral microbiologists, and materials and bioengineering experts in order to develop novel prevention technologies and implement the transfer of such technologies to industry and practice. Efforts to address the question of what can be done to more effectively use the large body of basic and applied caries science already available to speed solutions to the public health community should be the focus of future research initiatives.

## Competing interests

The author(s) declare that they have no competing interests.

## Authors' contributions

All authors read and approved the final manuscript.
